# Severe Discoloration of the Alveolar Bone Secondary to Long-Term Minocycline Therapy: A Case Report

**DOI:** 10.7759/cureus.64672

**Published:** 2024-07-16

**Authors:** Regina Friedl, Thomas Jackson, Thanhphuong Dinh, Thomas Y Yoon

**Affiliations:** 1 School of Dental Medicine, Lake Erie College of Osteopathic Medicine, Bradenton, USA

**Keywords:** dental esthetics, discoloration, alveolar bone, gingiva, minocycline

## Abstract

Minocycline, the synthetic derivative of the antibiotic tetracycline, has been used for a variety of medical treatments. One such use for minocycline is for acne vulgaris. Although widely used, minocycline has a common side effect of discoloration of tissues, including bone, skin, and mucosa. This case report presents a 19-year-old female patient with a history of long-term minocycline therapy for acne vulgaris who presented for periodontal esthetic crown lengthening. The initial exam revealed a blue-gray discoloration of the mucosa. Upon surgical exploration, it was discovered that the discoloration originated from the underlying alveolar bone with minimal gingival involvement. Surgical removal and recontouring of the bony exostoses revealed that the bone remained deeply stained. Although the discolored bone was not fully removed, the patient was able to obtain an acceptable esthetic result.

## Introduction

Tetracycline has been used for its therapeutic effects as a broad-spectrum antibiotic and antimicrobial since its introduction to the medical field in 1947. Shortly after tetracycline was commercialized for use as an antibiotic, findings surfaced that staining of bones and teeth could occur after prolonged use [1,2]. These early reports described deposits of tetracycline throughout the skeletal system, having a high affinity for the bones in the skull as well as teeth [2-7]. Other less common areas of tetracycline staining that have been identified include the ribs, vertebrae, femur, and humerus [4,8].

Minocycline is a synthetic derivative of the antibiotic tetracycline. Introduced in 1967, minocycline has been widely used for various medical conditions, including dermatologic treatment for chronic acne vulgaris. Preferential use of minocycline over tetracycline derivatives is attributed to its solubility in lipids, offering better infiltration into body fluids, high rate of absorption, and increased antimicrobial effects [9].

Minocycline antibiotic therapy has also been implicated in causing discoloration of skeletal elements via a deposition in these tissues [10-13]. Other tissues that are noted to be affected by minocycline staining are the skin, oral mucosa, tongue, nails, and extremities [14-18]. Such discoloration of skeletal elements is attributed to the secondary effect of minocycline chelation with calcium and magnesium [1,4]. Despite the effect of deposition into skeletal tissues, studies on this topic reveal that in adult bone, no deleterious effects occur to the structural integrity of the bone itself [7].

Staining of various tissues has been reported within the family of tetracyclines, including minocycline, since the 1950s. Reports of minocycline staining of maxillofacial skeletal elements are not novel; however, detailed clinical pictures with severity of staining are lacking. In this case report, a patient presents with severe blue-gray staining of alveolar bone that persists despite significant bone removal.

## Case presentation

**Figure 1:**
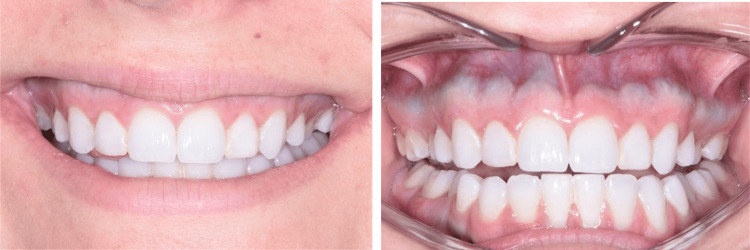
Intra-oral photos at the initial exam depict a natural smile (left). A band of blue-gray hue in the connective tissue is expanding past the mucogingival junction (right).

A 19-year-old Caucasian patient presented for an initial exam with the chief complaint of “a gummy smile." The patient was referred by her general dentist for esthetic crown lengthening to enhance the appearance of the front teeth. The initial exam revealed multiple buccal bony exostoses of both maxillary and mandibular alveolar bones. The bony exostoses extended across the full arch, with the greatest width at the level of the mucogingival junction (MGJ). Further examination revealed short maxillary clinical crowns with a dense band of keratinized tissue. The gingival tissues exhibited a color change from light pink to a blue/gray color as the keratinized gingiva reached the MGJ (Figure 1). The discoloration extended across most of the maxillary arch and between teeth numbers 20-29 on the mandibular arch (Figure 1). The dark pigmentation of the gingiva continued past the MGJ, extending 2-3 mm into the labial mucosa. A palatine torus was present, which also depicted the blue/gray hue showing through the palatal gingiva. The patient did not report any concerns with the color of her gingiva.

The patient’s medical history was significant for acne. The patient revealed no prior history of surgery or trauma. Medications included birth control and minocycline. Further investigation of her medical history revealed that the patient’s acne treatment was initiated in October 2014. The original medication prescribed was minocycline 100 mg capsule, twice daily (BID). On September 2017, the patient was instructed to decrease the dose to one 100-mg capsule per day. The initial minocycline treatment was completed in April 2018. Due to the recurrence of the acne, the patient started a second regimen of 100 mg of minocycline BID in August 2018 and was on this medication at the time of the periodontal treatment.

**Figure 2:**
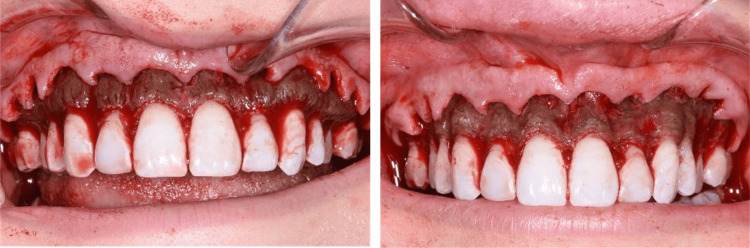
Surgical photos of the full-thickness reflected gingival flap reveal bony exostoses darkly pigmented with blue-gray alveolar bone (left). After 3 mm of bone was removed by crown lengthening, alveolar bone is still deeply pigmented (right).

The surgical treatment plan called for esthetic crown lengthening and bone reduction of the maxillary arch. Following adequate local anesthesia, a full-thickness mucoperiosteal flap was elevated. The maxillary alveolar bone revealed a generalized, darkly pigmented buccal bony exostosis (Figure 2). The reach of the discoloration extended across the entirety of the maxillary arch and was confined to bone only. A bone resection was performed to increase the length of the clinical crowns of the teeth. Additional recontouring of the facial maxillary bone was conducted to obtain a more harmonious gingival contour. The overall bone reduction was approximately 3-4 mm and was contoured to follow the roots of the teeth. Despite the bone reduction, the discoloration remained. Closure was obtained via interrupted sutures in each gingival papilla.

**Figure 3:**
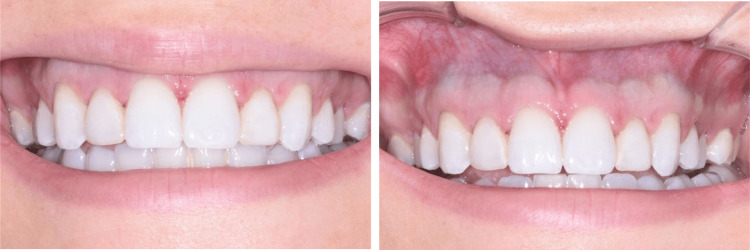
The patient presented for a one-week post-surgical exam with a natural smile (left). A retracted lip depicted the blue-gray pigmentation that was still present through the gingiva after the surgical healing period (right).

**Figure 4:**
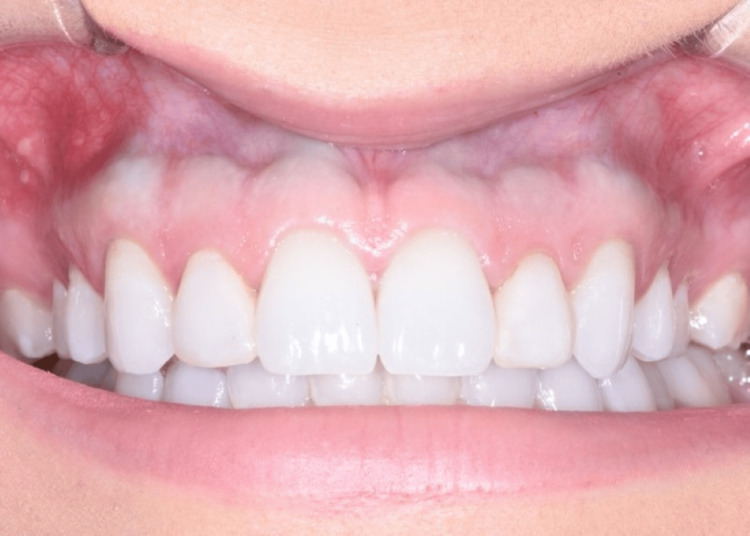
Retracted lip depicts the blue-gray pigmentation that is still visible through the gingival tissues at the level of the MGJ, although a marked decrease in pigmentation from pre-operative photos can be noted. MGJ: mucogingival junction

The patient returned in one week for a postoperative evaluation. There were no postoperative complications, and the patient healed uneventfully (Figure 3). A final postoperative appointment was conducted three months following the initial surgery. At this appointment, it was determined that the esthetic crown lengthening surgery significantly improved her chief complaint of a “gummy smile” (Figure 4). Further intra-oral investigation revealed that the discoloration of the gingiva was not fully removed, but significantly lessened. 

## Discussion

Long-term treatment with the antibiotics tetracycline and its derivatives, most notably minocycline, can deeply pigment tissues including skin, lips, teeth, oral mucosa, gingiva, and bone. Within the oral cavity, bone is the most common site of discoloration. Studies show that patients taking drugs such as minocycline have a 10% rate of bone discoloration after one year and as high as 20% after four years [16,19]. The patient in this case report was on a minocycline regimen for over four years and showed significant bone discoloration, which is consistent with current literature.

The benign discoloration in bones is caused by chelation of the antibiotic with calcium ions or short polypeptides and can be positively identified as a tetracycline-related compound by using Wood’s lamp [12]. As a result, depositions of tetracyclines and their derivates may incidentally lead to black bone discoloration. This case report identifies that the discoloration of bone is not just superficial and can extend deeper into skeletal structures. After 3-4 mm of osteoplasty, the bone continued to show a significant discoloration. Although thorough documentation of minocycline staining is available in the literature, there is minimal information regarding the extent of how deep minocycline staining can present within bone. Further studies should be conducted to investigate the depth of minocycline staining on bone. 

The clinical implications of minocycline discoloration are mostly esthetic in nature. Research shows that hyperpigmentation from minocycline does not affect bone integrity, and surgical procedures can be performed safely [20]. Animal studies show that minocycline increases osteoid surface, mineralizing surface, mineral apposition rate, bone formation rate, and reduced eroded surface in female rats [21]. 

To reduce the overall incidence of bone discoloration from minocycline, healthcare practitioners should consider alternative therapies for acne treatment. One such alternative treatment is the use of topical retinoids such as adapalene, tretinoin, and tazarotene. Retinoids are a diverse group of vitamin A derivatives that modulate gene expression and can regulate keratinocyte proliferation and differentiation [22]. Due to their effectiveness, topical retinoids are the preferred treatment and maintenance therapy for all acne, decreasing both comedonal and inflammatory acne lesion counts [23]. There is no report of bone staining from the use of topical retinoids.

If an oral antibiotic is the recommended treatment, alternative medications should be considered to replace minocycline. One such alternative is the macrolide antibiotic azithromycin. Macrolides inhibit protein synthesis by binding the 50S subunit of the ribosome [24]. Azithromycin is a derivative of erythromycin, which was once used as an effective acne therapy [25]. Azithromycin is used to treat serious systemic infections, and its use for acne is generally reserved for select cases, such as in patients for whom tetracyclines are contraindicated [26].

If minocycline is the preferred drug of choice, considerations should be made to reduce the incidence of bone staining. Bernier and Dreno stated that a reduction in dosage from 100 mg a day to 50 mg a day after 15 days was effective at preventing bone staining [27]. 

The gingival thickness in this case may have helped to reduce the overall discoloration. Jung et al. investigated the relationship between gingival thickness and the color of restorative materials. It was concluded that an increase in gingival thickness reduced the discoloration due to restorative materials [28]. In this case, the patient had a thick gingival biotype with adequate keratinized tissue. Her tissue thickness may have helped to mask the underlying discolored bone. 

Future research should be done to explore the depth of minocycline staining in bone and potential mechanisms to reduce overall staining. One area that has shown promise in the reduction of bone staining is the administration of vitamin C while taking minocycline. Vitamin C has been shown to decrease the formation of the quinine ring structure which is a component of the minocycline stain [29]. This study was performed on an animal model, and further investigation should be conducted.

Interprofessional collaboration between dentists, physicians, and pharmacists can provide a comprehensive approach to managing and preventing minocycline-induced discoloration of bones. Physicians can collaborate with dentists to determine if staining could present an esthetic concern. Patients with a low smile line that does not show excessive gingiva may not be as concerned with staining and may be better candidates for minocycline therapy. Dentists and pharmacists should have a conversation with patients before starting minocycline treatment to educate them on the possible effects of bone staining. This interprofessional collaboration will help to provide better outcomes for patients being treated with minocycline for acne.

## Conclusions

Minocycline is an antibiotic that is commonly used for the treatment of acne-related disorders. A side effect of the utilization of minocycline is discoloration of the bones. The maxillary and mandibular bones show the highest amount of discoloration among all structures in the mouth. This discoloration can affect the gingiva and result in unesthetic consequences. Dental practitioners should be aware of this phenomenon and plan treatment cases accordingly to reduce the effect of gingival discoloration due to minocycline-stained alveolar bone.
